# Extreme mitochondrial variation in the Atlantic gall crab *Opecarcinus hypostegus* (Decapoda: Cryptochiridae) reveals adaptive genetic divergence over *Agaricia* coral hosts

**DOI:** 10.1038/srep39461

**Published:** 2017-01-12

**Authors:** Kaj M. van Tienderen, Sancia E. T. van der Meij

**Affiliations:** 1 Naturalis Biodiversity Center, Darwinweg 2, 2333 CR Leiden, the Netherlands; 2 Oxford University Museum of Natural History, Parks Road, Oxford OX1 3PW, United Kingdom; 3 Linacre College, St Cross Rd, Oxford OX1 3JA, United Kingdom

## Abstract

The effectiveness of migration in marine species exhibiting a pelagic larval stage is determined by various factors, such as ocean currents, pelagic larval stage duration and active habitat selection. Direct measurement of larval movements is difficult and, consequently, factors determining the gene flow patterns remain poorly understood for many species. Patterns of gene flow play a key role in maintaining genetic homogeneity in a species by dampening the effects of local adaptation. Coral-dwelling gall crabs (Cryptochiridae) are obligate symbionts of stony corals (Scleractinia). Preliminary data showed high genetic diversity on the COI gene for 19 *Opecarcinus hypostegus* specimens collected off Curaçao. In this study, an additional 176 specimens were sequenced and used to characterize the population structure along the leeward side of Curaçao. Extremely high COI genetic variation was observed, with 146 polymorphic sites and 187 unique haplotypes. To determine the cause of this high genetic diversity, various gene flow scenarios (geographical distance along the coast, genetic partitioning over depth, and genetic differentiation by coral host) were examined. Adaptive genetic divergence across Agariciidae host species is suggested to be the main cause for the observed high intra-specific variance, hypothesised as early signs of speciation in *O. hypostegus*.

A central challenge in evolutionary biology is to establish the influence of spatial and ecological processes on the evolutionary patterns of species, including local adaptation, colonization and speciation^1^,^2^. Gene flow is the genetically effective exchange of migrants among populations^3^, depending on the rate of exchange and the migrants’ fitness^4^. Patterns of gene flow have a strong effect on the evolution of a species by dampening the genetic response to local selection, as they tend to make gene frequencies uniform among populations, whereas genetic drift and adaptation tend to diversify populations^4^,^5^,^6^. It is easy to comprehend how in a terrestrial environment the landscape (e.g. mountains, rivers or forests) can act as a barrier to gene flow, and give rise to genetic divergence between conspecific populations. Understanding how genetic differentiation arises in a marine landscape is, however, a more challenging task^7^. Consequently, the patterns of gene flow remain understudied for many marine species^2^,^8^,^9^,^10^. Genetic methods are powerful tools to examine genetic connectivity among individuals and to determine the spatial population structure of marine species^10^,^11^,^12^,^13^.

The population genetic structure in marine species can be affected by several mechanisms. Gene flow patterns may be proportional to geographic distance, whereby genetic differentiation increases with distance^11^. Although oceanic currents can have a homogenizing effect on the genetic structure of populations, other geographical factors such as habitat discontinuity, local current systems and physical barriers can act as limitations to gene flow^14^. Then again, gene flow may be higher among ecologically similar environments^6^.

Many marine invertebrates exhibit a pelagic larval stage. The effectiveness of migration is determined by the duration of the pelagic larval phase and the strength of oceanic currents, together affecting the realized larval dispersal distance, as well as factors such as the survival and reproduction rate of the successfully dispersed larvae in a novel habitat^15^. Because pelagic larvae can potentially disperse both horizontally and vertically, ecological differences over depth gradients, such as light, temperature and turbidity, may also give rise to different selection pressures resulting in genetic diversification in a marine environment^16^,^17^. Correlations between genetic differentiation and depth distances have been measured for various corals; for instance, *Pocillopora damicornis* (Linnaeus, 1758)^18^ and Atlantic Agariciidae corals^19^.

The importance of environmental factors on the genetic structuring of populations has been shown in marine species^20^, but the effect of these factors on gene flow over a small geographical range has received little attention so far^21^. Furthermore, active habitat selection, for example in organisms restricted to a particular habitat (such as associated organisms), could act as a barrier to dispersal. Coral-dwelling gall crabs (Cryptochiridae) are obligate symbionts of stony corals (Scleractinia), and display high degrees of host specificity^22^,^23^,^24^. Their larvae settle on corals as a megalopae and modify coral morphology by inducing the growth, or possibly excavation, of pits or galls^25^,^26^,^27^,^28^. Female gall crabs reside for a lifetime in these dwellings, whereas male gall crabs either inhabit a dwelling or are found to be free-living^29^. Larval development is scarcely known for Cryptochiridae, but is thought to consist of at least five, and possibly seven, planktonic larval stages^30^. In a study on the host species of Atlantic gall crabs, 19 specimens of *Opecarcinus hypostegus* (Shaw and Hopkins, 1977) were collected off Curaçao^29^. *Opecarcinus hypostegus* is associated with five *Agaricia* species and *Helioseris cucullata* (Ellis and Solander, 1786) of the family Agariciidae^29^,^31^,^32^. Interestingly, the observed depth range of *O. hypostegus* includes very shallow as well as deeper reefs down to at least 60 m^33^. Transect data at 6 m, 12 m, and 18 m revealed a depth preference in *O. hypostegus* for the deeper reefs. Prevalence rates at 6 m were highest in *Agaricia agaricites* (Linnaeus, 1758) and at 12 m and 18 m highest in *Agaricia lamarcki* Milne Edwards and Haime, 1851^34^. High genetic diversity was observed at the cytochrome-c oxidase I (COI) gene for the 19 collected specimens obtained from different localities along the Curaçaoan coast, from various depths and five *Agaricia* coral hosts. Seventy-six polymorphic sites, resulting in a nucleotide diversity (*π*) of 0.02617 and a haplotype diversity (*h*) of 1.00 were retrieved (van der Meij, unpubl. data). These results were surprising, because most Indo-Pacific members of the Cryptochiridae show very low haplotype diversity at the COI gene across large distances and COI is most commonly used to infer phylogenetic relationships at species level^24^,^35^,^36^ but see ref. 37.

The purpose of this study is to examine the possible barriers that affect the genetic structure of *O. hypostegus* in more detail. COI sequence data was used to characterize *O. hypostegus* population structure and infer patterns of *O. hypostegus* gene flow along the leeward side of Curaçao. Factors that are expected to limit gene flow and increase genetic differentiation at this small geographical scale include: (I) geographical distance along the leeward side of Curaçao, (II) genetic partitioning over depth, or (III) genetic differentiation between individuals inhabiting different *Agaricia* species.

## Results

### Patterns of polymorphism

A 675 base pairs long fragment of the COI region was sequenced for a total of 195 individuals (Table S1). Across all collection sites, 146 nucleotide sites were polymorphic, yielding 187 unique haplotypes (*h* = 0.9994). Of these, 123 were third codon position changes, along with 23 first codon position changes and zero second codon position changes. Overall nucleotide diversity (*π*) = 0.02558 (Table 1). Translation of the sequences to amino acid data revealed only five polymorphisms in five individuals (RMNH.Crus.D.57581, 57456, 57557, 57559 and 57476; Table S1), all at different positions of the sequence. An Automatic Barcode Gap Discovery (ABGD) analysis shows that only one Molecular Operational Taxonomic Unit is present in *O. hypostegus.*

### Population structure

#### Geographic differentiation

**Figure 1:**
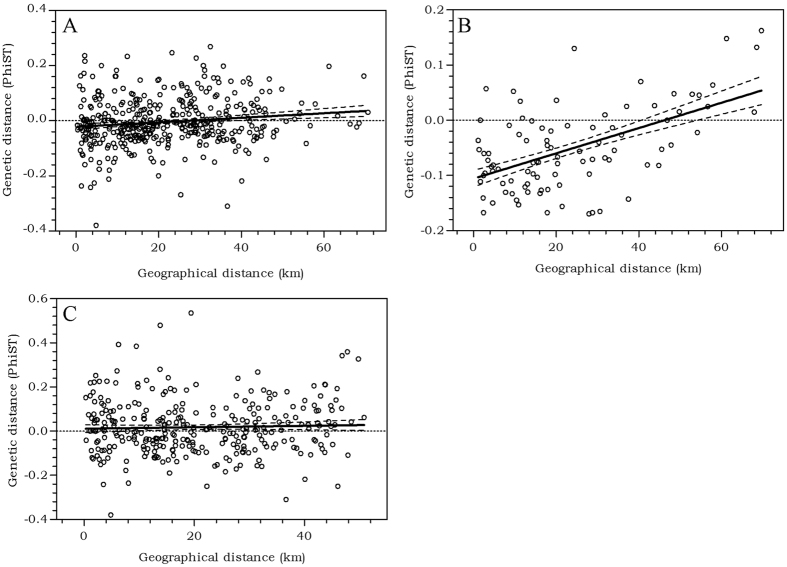
Genetic differentiation over geographical distance, shown as the regression of pairwise Φst between populations (localities) against the distance (km). (**A**) All *Agaricia* coral hosts (r = 0.1408, P = 0.0587), linear regression slope = 0.0008263 ± 0.0002042 (P < 0.0001) and R^2^ = 0.01982; (**B**) individuals inhabiting *A. agaricites* (r = 0.5439, P = 0.0022), linear regression slope = 0.002290 ± 0.0002634 (P < 0.0001) and R^2^ = 0.2959; (**C**) individuals inhabiting *A. lamarcki* (r = −0.0356, P = 0.3085), linear regression slope = 0.0003268 ± 0.0003756 (P = 0.3846) and R^2^ = 0.001264.

A Mantel test revealed an isolation-by-distance pattern off Curaçao for *O. hypostegus*, with a relationship between the genetic differentiation (Φst) (Table S2) and the geographic distance (km) between the collection sites (Table S3) (r = 0.1408, P = 0.0587) (Fig. 1A). Partitioning the Isolation By Distance (IBD) analysis into groups of individuals collected from the same agariciid coral species, *Agaricia lamarcki* (n = 117), *Agaricia agaricites* (n = 66), *Agaricia humilis* Verrill, 1901 (n = 7), *Agaricia grahamae* Wells, 1973 (n = 4) or *Agaricia fragilis* Dana, 1846 (n = 1), revealed a significant relationship between genetic differentiation (Φst) and geographic distance (km) for individuals sampled from *A. agaricites* (r = 0.5439, P = 0.0022) (Fig. 1B). No significant relationship between genetic differentiation (Φst) and geographic distance (km) was found, however, for individuals sampled from *A. lamarcki* (r = −0.0356, P = 0.3085) (Fig. 1C). For the individuals sampled from the remaining host coral species, *A. humilis, A. grahamae* and *A. fragilis*, population sample sizes were too small or too few populations were sampled to perform a valid IBD analysis (Table S1).

#### Depth differentiation

**Figure 2:**
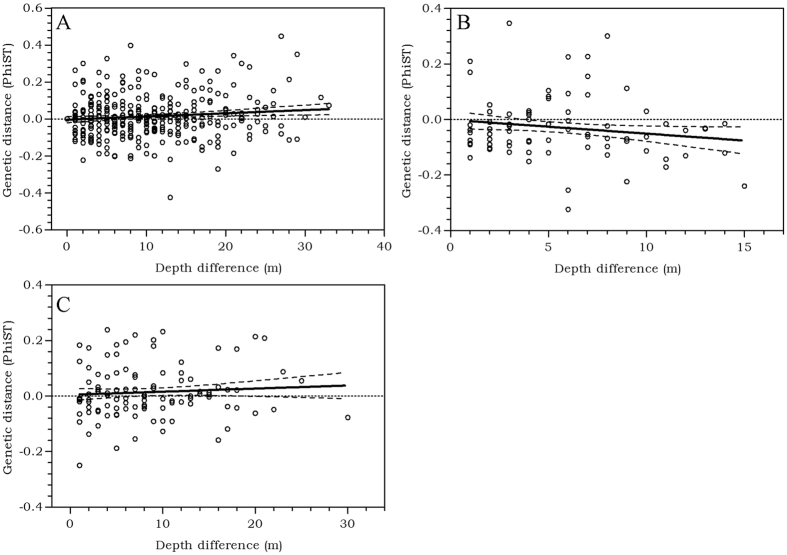
Genetic differentiation over depth, shown as the regression of pairwise Φst between populations (collection depths) against the depth difference (m). (**A**) All *Agaricia* coral hosts, (r = 0.1063, P = 0.1426), linear regression slope = 0.001723 ± 0.0006580 (P = 0.0090) and R^2^ = 0.01132; (**B**) individuals inhabiting *A. agaricites* (r = −0.1604, P = 0.8599), linear regression slope = −0.004988 ± 0.002473 (P = 0.0454) and R^2^ = 0.02574; (**C**) individuals inhabiting *A. lamarcki* (r = 0.0465, P = 0.3372), linear regression slope = 0.001101 ± 0.001079 (P = 0.3090) and R^2^ = 0.004976.

A mantel test was used to test for a relationship between genetic differentiation (Φst) and the difference in depth of collection (Table S1) between each sample, but no significant relationship was found (r = 0.1063, P = 0.1426). Hence, there is no statistical evidence for genetic isolation over depth (Fig. 2A). Partitioning the IBD analysis into individuals collected from the same host coral species had no effect on the outcome. No significant relationship was found between genetic differentiation (Φst) and depth for individuals sampled from *A. agaricites* (r = − 0.1604, P = 0.8599) (Fig. 2B), nor for individuals sampled from *A. lamarcki* (r = 0.0465, P = 0.3372) (Fig. 2C). For the individuals sampled from the remaining host coral species, *A. humilis, A. grahamae* and *A. fragilis*, population sample sizes were too small or too few populations were sampled to perform a valid IBD analysis (Table S1).

#### Genetic subdivision between individuals collected from different host corals

**Figure 3:**
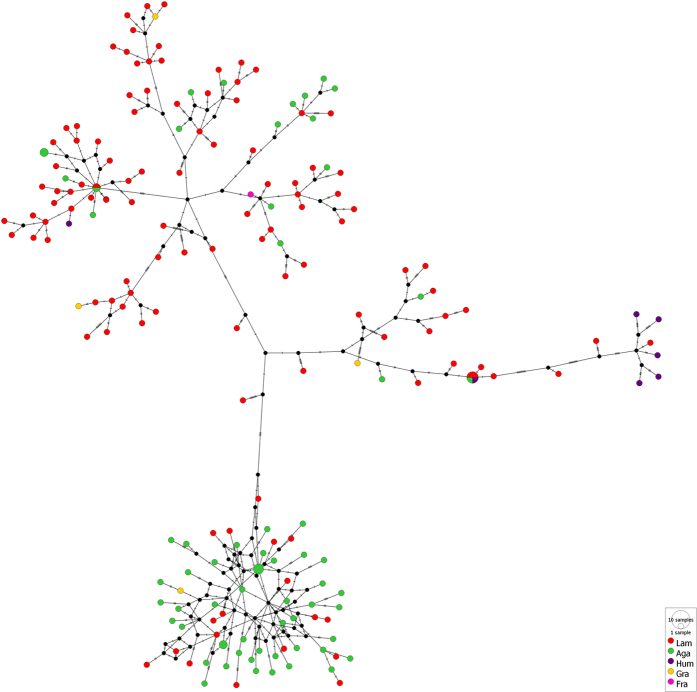
Median joining haplotype network showing the mutational distance between *O. hypostegus* individuals. Colours indicate coral host species: in red *A. lamarcki* (Lam), in green *A. agaricites* (Aga), in purple *A. humilis* (Hum), in yellow *A. grahamae* (Gra) and in pink *A. fragilis* (Fra), see legend. Number of mutation steps is shown as hatch marks.

**Figure 4:**
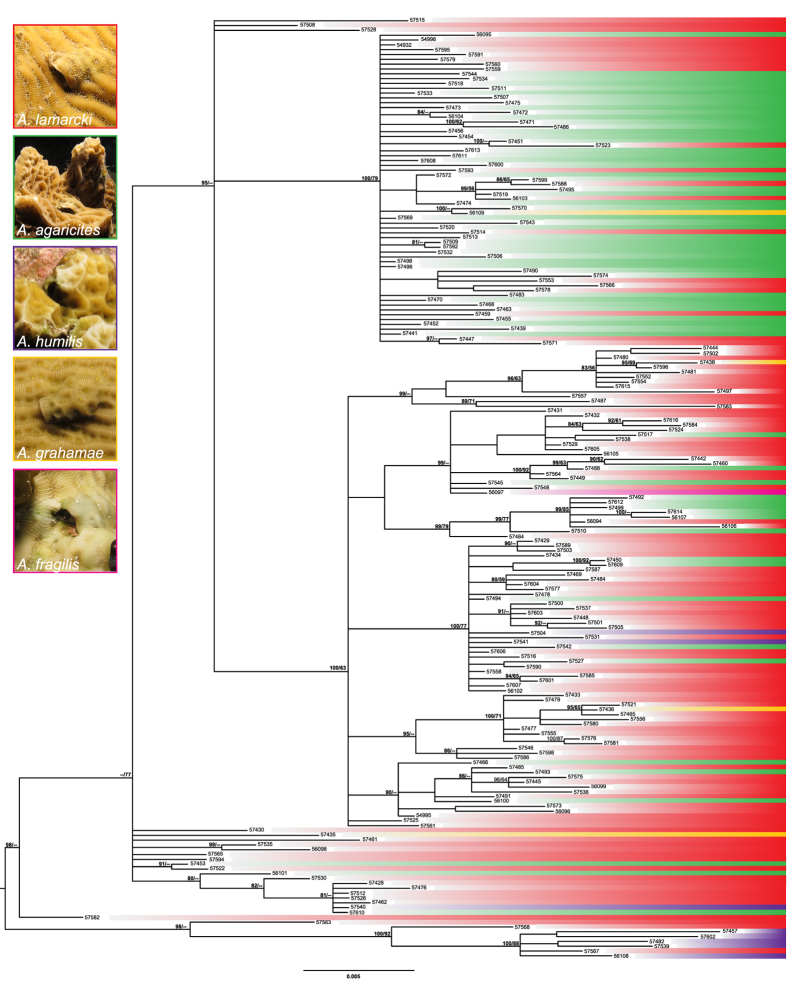
Phylogram of *O. hypostegus* based on the Bayesian consensus tree showing Bayesian inference support values (left) and maximum likelihood bootstrap support values (right). The numbers refer to the collection numbers of the material (Table S1), the colours indicate the coral host species and correspond with those in Fig. 3.

Fu and Li’s F, and Tajima’s D were negative for the individuals sampled from the host coral species *A. lamarcki, A. agaricites* and *A. grahamae*, but not statistically significant (Table 1). An Analysis of MOlecular VAriance (AMOVA) indicated statistically significant genetic differentiation between individuals sampled from different host coral species (P < 0.00001) (Figs 3 and 4, Table 2). Pairwise Fst’s indicate significant genetic differentiation between the *O. hypostegus* individuals collected from different host coral species (Table 3). The strongest genetic differentiation was measured between *A. humilis* and *A. agaricites* (Fst 0.38309, P < 0.0001), followed by *A. humilis* and *A. lamarcki* (Fst 0.26133, P < 0.0001), *A. agaricites* and *A. lamarcki* (0.15726, P < 0.0001) and *A. agaricites* and *A. grahamae* (Fst 0.15070, P = 0.03604). Negligible differentiation was measured between *A. grahamae* and *A. lamarcki* (Fst 0.09234, P = 0.02703) (Table 3). No significant genetic differentiation was measured between individuals collected from *A. grahamae* (n = 4) and *A. humilis* (n = 7), possibly due to the small samples sizes. These results are in concordance with the median joining network (Fig. 3) that reveals a large mutation distance between individuals inhabiting *A. humilis* or *A. agaricites* and individuals collected from the other host corals. The largest mutation distance was found between *O. hypostegus* individuals inhabiting the coral hosts *A. humilis* and *A. agaricites* (Fig. 3). The groupings in the phylogenetic tree (Fig. 4) are in agreement with those of the haplotype network. The clade with *O. hypostegus* inhabiting *A. humilis* is retrieved with high support values and relatively long branch lengths. Within the large overall clade there are a few singletons, which are those individuals clustering closest to *A. humilis* in the haplotype network. Within the clade mostly associated with *A. agaricites*, little clustering is observed, which can be linked to the starlike structure in the network. Within the clade associated with *A. lamarcki*, more clustering is observed. The individuals inhabiting *A. grahamae* and *A. fragilis* are retrieved in various parts of the haplotype network and phylogenetic tree (Figs 3 and 4).

## Discussion

### Patterns of polymorphism

In the COI sequence data of the 195 *Opecarcinus hypostegus* specimens collected from Curaçao, 146 COI polymorphic sites were found and 187 unique haplotypes (Table 1). Strikingly, in Cryptochiridae collected from various locations in the Indo-Pacific hardly any polymorphic sites are present on the COI gene, even over distances as large as between the Indo-Malayan region and New Caledonia^24^, or the Red Sea and Japan^36^. Generally, COI sequence data shows high resolution at species level, and work well as a barcoding marker.

First and second COI codon positions are highly conserved, whereas third codon positions can evolve rapidly, making this locus a common choice for population genetics and phylogeography^38^,^39^. As expected, almost all variation in *O. hypostegus* was found on the third codon position. When translated to amino acids, however, only five polymorphisms at five different positions were retrieved, hence there are no cryptic species present in *O. hypostegus.* This result was confirmed by the ABGD analysis. Extreme levels of genetic variation have been reported within natural populations in, for example, planktonic chaetognaths (arrow worms)^40^ and mesopelagic shrimp^41^. Many instances of high mitochondrial diversity have directly or indirectly been interpreted as evidence of cryptic speciation, but some of these cases may need to be subjected to re-evaluation when investigated using nuclear loci^40^,^42^.

The genetic diversity obtained in this study (mean *h* = 0.9994, mean *π* = 0.02558), from a very small geographic area, can be classified as an extreme level of intra-specific variance compared to the reported mean and median values for haplotype (0.63388 and 0.70130) and nucleotide diversity (0.00388 and 0.00356) for 23 animal species in a meta-analysis^43^, which showed a positive, non-linear relationship between the population-level estimates of *h* and *π*. The values obtained in our study strongly deviate from their values, with *π* being much higher. A combination of high nucleotide and haplotype diversities has been linked to large stable populations with a long evolutionary history and possible secondary contact between differentiated lineages^44^. In contrast, the negative indexes of neutrality Fu and Li’s F, and Tajima’s D indicate a departure from neutral processes, which can be caused by demographic changes or selective events. Due to the non-significance of these values the hypothesis of neutrality can, however, not be rejected.

### Small scale geographical genetic differentiation

The leeward side of Curaçao is about 65 km long from southeast to northwest. For the seaweed *Sargassum polyceratium* Montagne, 1837, fine-scale differentiation was retrieved around Curaçao, with bays showing significant differentiation from each other^21^. A Mantel test revealed a relationship between the genetic similarity of certain individuals and geographical distance for *O. hypostegus* (Fig. 1A). Although the relationship was weak and not highly significant, this suggests a genetic structure within *O. hypostegus* individuals living in close spatial proximity being more genetically similar than expected under a random distribution of genotypes. Splitting the sample into groups of individuals collected from the same host coral species increased both the magnitude and significance of the isolation-by-distance pattern for individuals inhabiting *Agaricia agaricites* (Fig. 1B). For *A. lamarcki*, no statistical evidence for isolation-by-distance was retrieved (Fig. 1C). This difference may be explained by the higher abundance of *A. agaricites* corals off Curaçao, compared to *A. lamarcki*^34^, providing suitable habitat closer to the natal site of the *O. hypostegus* larvae settling on *A. agaricites*. Furthermore, *A. lamarcki* has a wider depth distribution than *A. agaricites*^19^, which may influence the isolation-by-distance results.

### Genetic partitioning over depth

No statistical evidence was found for genetic differentiation over depth in *O. hypostegus* (Fig. 2A), at least not within the studied depth range of this study (5–38 m). Partitioning the analysis into groups of individuals collected from the same host coral had no effect on the outcome, and individuals inhabiting *A. agaricites* or *A. lamarcki* did not show any significant genetic differentiation over depth (Fig. 2B,C).

Due to the technical limitations of scientific diving, our sampling was restricted to a maximum of 38 m depth. The depth distribution of *O. hypostegus* is, however, known to extend to the mesophotic zone (ca. 60 m), where an *O. hypostegus* individual was observed inhabiting an *A. lamarcki* coral^33^. We may argue that sampling over a depth range that is at least twice as large as in the present study, would reflect the total *O. hypostegus* distribution more completely. This could increase the likelihood of revealing genetic differentiation over depth, because sampled individuals living over a larger depth distribution face more variable environmental conditions.

### Genetic differentiation over coral hosts

Under an ecology-driven gene flow scenario, gene flow may be strongest among similar environments^6^,^45^,^46^,^47^,^48^,^49^. This pattern may arise through mechanisms such as selection and local adaptation that will disrupt the patterns of isolation-by-distance^50^ or as a consequence of selection against maladapted immigrants from different environments^6^. In our study, significant genetic subdivisions between individuals inhabiting different host coral species were observed (Tables 2 and 3; Figs 3 and 4). There was statistical evidence for diversification across host coral species (i.e. environment) in *O. hypostegus*, which is expected to be an important alternative strategy to direct competition for the same host in Cryptochiridae^23^.

Cryptochirid males “visit” females inhabiting separate galls or pits, dubbed the “visiting” mating system^29^,^51^. It is unclear how far a male gall crab can travel to find a female partner. Many male and female gall crabs can inhabit the same coral colonies, especially if these are large in size. If a male *O. hypostegus* mates with females on the same coral colony, a genetic preference for that coral host might end up getting fixed in a population. The study on gall crab occurrence rates on the leeward side of Curaçao revealed significant higher *O. hypostegus* prevalence in *A. lamarcki* compared to *A. agaricites* and *A. humilis*, suggesting a preference for inhabiting *A. lamarcki*^34^.

Phylogenetic results showed in the median joining network (Fig. 3) and phylogenetic tree (Fig. 4) support the genetic differentiation across host species in *O. hypostegus*, with distinct clustering of individuals inhabiting the hosts *A. lamarcki, A. agaricites* and *A. humilis*. In the haplotype network, the observed groupings show different patterns. A star-shaped burst pattern (interlinked haplotypes with few mutation steps between them) can be observed for the individuals inhabiting *A. agaricites* (Fig. 3)^44^. These patterns appear due to high numbers of low frequency alleles with small average pair-wise distances between them, and may be evidence of a recent expansion from a small number of ancestors. In addition, the nucleotide diversity of specimens inhabiting *A. agaricites* is lower in comparison to other coral hosts (Table 1). In an expanding population, haplotype diversity and number of polymorphic sites can quickly increase, while nucleotide diversity usually lags behind. Indeed, high levels of *h* with moderate to low levels of *π* have frequently been attributed to recent divergence in marine species^52^. Over time, when a population stops expanding and starts to stabilize, nucleotide diversity will increase. Newly created low frequency haplotypes either increase in the population or are lost, which increases the average number of segregating sites between haplotypes over time^44^. In the *A. lamarcki* grouping, a higher number of segregating sites is observed between the haplotypes (Fig. 3), suggesting that this population is stabilizing.

A study on the historical evolutionary patterns of cryptochirids has indicated that the phylogeny of coral gall crabs is directed by the evolution of their scleractinian hosts^23^. For yet unknown reasons, Indo-Pacific gall crab species show stricter host-specificity patterns than their Atlantic counterparts^33^. The congeners of *O. hypostegus* in the Indo-Pacific are highly host specific and are often associated with one or several closely related coral species^53^,^54^. Presumably, the current genetic diversification across host corals found in *O. hypostegus* may not only result in a stronger local-adaptation to ecological differences between coral hosts over time, but might even be strong enough to eventually foster speciation.

## Concluding remarks

The main objective of the present study was to examine which spatial and ecological factors influence *Opecarcinus hypostegus* gene flow off Curaçao and can explain the observed high genetic diversity at the COI gene. Factors that were expected to influence the *Opecarcinus hypostegus* gene flow patterns were examined; we found a weak relationship for geographical genetic differentiation (mostly for the host coral *A. agaricites*) and no evidence for genetic differentiation over depth. The observed clustering in the haplotype network and phylogenetic tree, however, suggests that adaptive divergence over the coral hosts is present. We hypothesise that this divergence is an early sign of (sympatric) speciation. This divergence might result in several closely related species of *Opecarcinus* inhabiting *Agaricia* corals in the Caribbean, in a similar way to its congeners inhabiting various closely related Agariciidae corals in the Indo-Pacific^53^,^54^. To further test this hypothesis, data from additional markers and localities is needed.

## Materials and Methods

### Field sampling

During field surveys in 2013 (16 Oct–9 Nov) and 2014 (12 Mar–28 Apr), specimens of *Opecarcinus hypostegus* were collected by chiselling off a small piece of their agariciid coral host from depths between 5 and 38 m at 29 localities on the leeward side of Curaçao (Dutch Caribbean, southern part of the Caribbean Sea). Four samples were collected at ca. 20 m depth from the island Klein Curaçao, located approximately 10 kilometres southeast of Curaçao (Fig. S1, Table 4, S1). The corals were visually identified to species level during the surveys using field guides, Coralpedia (http://coralpedia.bio.warwick.ac.uk) and the Coral IDC tool (http://www.researchstationcarmabi.org)^55^. The leeward side of Curaçao stretches some 65 km from southeast to northwest with an almost continuous coral reef, providing uninterrupted suitable habitat for gall crab larvae settlement in which no clear geographical barriers appear to occur. In total, 210 *O. hypostegus* gall crab samples were collected from five *Agaricia* host coral species. Crabs were preserved in ethanol (80% in 2013, 96% in 2014). All collected specimens are deposited in the Crustacea collection of the Naturalis Biodiversity Center in Leiden (formerly Rijksmuseum van Natuurlijke Historie, collection coded as RMNH.Crus.D).

### DNA analyses

For 195 specimens, the DNA was isolated from muscle tissue of the fifth pereiopod using the NucleoMag 96 Kit (Machery-Nagel) according to the manufacturer’s protocol for animal tissue. The coral hosts of these 195 individuals were: *Agaricia lamarcki* (n = 117), *A. agaricites* (n = 66), *A. humilis* (n = 7), *A. grahamae* (n = 4) and *A. fragilis* (n = 1). Polymerase chain reaction was carried out with the following conditions; PCR CoralLoad Buffer (containing 15 mM MgCl_2_), 0.5 *μ*L dNTPs (2.5 mM), 1.0 *μ*L of each primer, LCO-1490 and HCO-2198^56^, 0.3 *μ*L Taq polymerase (15 units per *μ*L), 18.7 *μ*L of extra pure PCR water and 1.0 *μ*L DNA template. Thermal cycling was performed by initial denaturation at 95 °C for 3 min, followed by 39 cycles of 95 °C for 10 s, 48 °C for 1 min and 72 °C for 1 min, and a final elongation step of 5 min at 72 °C. PCR products were sequenced by BaseClear BV (Leiden, The Netherlands). Sequences were assembled and edited in Sequencher^®^ v. 5.3 (Gene Codes Corporation, Ann Arbor, MI USA) and aligned with ClustalW in BioEdit 7.2.5^57^. Sequences are deposited in GenBank under accession numbers KU041838, and KY026220-KY026413 (Table S1).

### Molecular analyses

The number of polymorphisms (P), nucleotide diversity (*π*), haplotype diversity (*h*), Fu and Li’s F and Tajima’s D values were calculated using DnaSP 5.10.01^58^. Nucleotide diversity is defined as the average number of nucleotide differences per site between two randomly chosen DNA sequences^59^. Haplotype diversity is defined as the probability that two randomly chosen haplotypes are different^60^. The Fu and Li’s F, and Tajima’s D values are used to determine whether the population evolves neutrally^61^,^62^.

Prior to the model-based phylogenetic analysis, the best-fit model of nucleotide substitution was identified for each gene partition by means of the corrected Akaike Information Criterion (AICc) calculated with MEGA 6.06^63^, resulting in GTR+I+G as the most suitable model of nucleotide substitution. A maximum likelihood analysis (GTR+I+G; 1000 bootstraps) was carried out with Phyml 3.1^64^ using the Seaview platform^65^. Bayesian inferences coupled with Markov chain Monte Carlo techniques (six chains) were run for 5,000,000 generations in MrBayes 3.2.6^66^, with a sample tree saved every 1000 generations and the burnin set to 25%. Consensus trees were visualized in FigTree v.1.3.1.

A median joining haplotype network, to display the COI sequence variation^67^, was build using PopART 1.7 (http://popart.otago.ac.nz). For continuously distributed populations a Mantel’s test between the genetic differentiation and geographical distance can detect an isolation-by-distance pattern. A Mantel’s test was performed in the program IBDWS v. 3.23^68^ using Φst as a measure of genetic differentiation, which incorporates sequence distance information. Significance was determined by permuting the data 30,000 times. The population structure was described with an analysis of molecular variance method (AMOVA)^69^ implemented in ARLEQUIN^70^. Significance was determined with 10,000 random permutations of the data. Arlequin was also used to calculate the pairwise Fst values between individuals collected from different host coral species.

The web version of ABGD^71^ was used to estimate the genetic distance corresponding to the difference between a speciation process versus intra-specific variation in *O. hypostegus*. Runs were performed using the default range of priors (pmin = 0.001, pmax = 0.10) using the JC69 Jukes-Cantor measure of distance. The analysis involved 195 sequences with a total of 675 positions in the final dataset.

## Additional Information

**How to cite this article**: van Tienderen, K. M. and van der Meij, S. E. T. Extreme mitochondrial variation in the Atlantic gall crab *Opecarcinus hypostegus* (Decapoda: Cryptochiridae) reveals adaptive genetic divergence over *Agaricia* coral hosts. *Sci. Rep.* **7**, 39461; doi: 10.1038/srep39461 (2017).

**Publisher's note:** Springer Nature remains neutral with regard to jurisdictional claims in published maps and institutional affiliations.

## Supplementary Material

Supplementary Fig. S1

Supplementary Dataset 1

Supplementary Dataset 2

Supplementary Dataset 3

## Figures and Tables

**Table 1:** Number of polymorphisms (P), number of unique haplotypes (H), indexes of neutrality Fu and Li’s F, and Tajima’s D, haplotype diversity (*h*) and nucleotide diversity (*π*) for *O. hypostegus* individuals sampled from each of the agariciid host coral species based on COI sequence data.

Coral host	N	P	H	Tajima’s D	Fu and Li’s F	*h*	*π*
All corals	195	146	187	−1.28229*	−1.95521*	0.9994	0.02558
*Agaricia lamarcki*	117	127	115	−1.35254*	−2.29146*	0.9996	0.02400
*A. agaricites*	66	96	62	−1.25541*	−1.53289*	0.9980	0.02057
*A. humilis*	7	44	7	−0.17519*	0.05205*	1.0000	0.02638
*A. grahamae*	4	36	4	−0.69771*	−0.59275*	1.0000	0.02864
*A. fragilis*	1	—	—	—	—	—	—

The values for *A. humilis* and *A. grahamae* are based on low numbers. ^*^Not significant (P > 0.05).

**Table 2:** Analysis of molecular variance (AMOVA) among and within the groups of *O. hypostegus* individuals sampled from the agariciid host coral species, *A. lamarcki, A. agaricites, A. humilis, A. grahamae* or *A. fragilis*.

Source of variation	df	Sum of squares	Variance component	%	P-value
among host species	3	189.236	1.689	17.49	< 0.00001
within host species	191	1522.359	7.970	82.51	< 0.00001

**Table 3:** Matrix of the pairwise Fst’s indicating the magnitude of genetic differentiation between the groups of *O. hypostegus* individuals sampled from the agariciid host coral species, *A. lamarcki, A. agaricites, A. humilis, A. grahamae* or *A. fragilis*.

	*Agaricia lamarcki*	*A. agaricites*	*A. humilis*	*A. grahamae*
*Agaricia lamarcki*	0	0.15726 (p < 0.0001)	0.26133 (p < 0.0001)	0.09234 (p = 0.02703)
*A. agaricites*	0.15726 (p < 0.0001)	0	0.38309 (p < 0.0001)	0.15070 (p = 0.03605)
*A. humilis*	0.26133 (p < 0.0001)	0.38309 (p < 0.0001)	0	0.10726 (p = 0.10811)
*A. grahamae*	0.09234 (p = 0.02703)	0.15070 (p = 0.03605)	0.10726 (p = 0.10811)	0

**Table 4:** Detailed sampling locality data including: locality codes, site name, collection site coordinates, number of collected samples (N), number of polymorphic sites (P) and nucleotide diversity (π) per locality.

Locality	Site name	Latitude	Longitude	n	P	*π*
CAR01	Piscadera Bay	N 12°07.329′	W 068°58.131′	9	50	0.02617
CAR02	Waterfabriek	N 12°06.588′	W 068°57.241′	2	24	0.03556
CAR03	Sea Aquarium	N 12°04.907′	W 068°53.763′	2	14	0.02074
CAR04	Blue Bay	N 12°08.063′	W 068°59.138′	3	24	0.02420
CAR05	Playa Kalki	N 12°22.536′	W 069°09.464′	5	35	0.02341
CAR06	Superior Producer	N 12°06.354′	W 068°56.572′	3	28	0.02765
CAR07	Kleine Knip	N 12°20.475′	W 069°09.154′	8	56	0.03058
CAR09	Sunset Waters	N 12°16.046′	W 069°07.657′	4	38	0.02963
CAR10	Playa Lagun	N 12°19.090′	W 069°09.036′	4	39	0.03136
CAR11	Playa Manzalina	N 12°14.713′	W 069°06.314′	14	52	0.02203
CAR12	Caracasbaai	N 12°04.588′	W 068°51.817′	3	24	0.02370
CAR13	Jan Thiel	N 12°04.584′	W 068°52.796′	4	35	0.02765
CAR14	Vaersenbaai	N 12°09.678	W 068°00.237′	7	47	0.02787
CAR15	Klein Curaçao	N 11°59.087	W 068°38.662′	4	31	0.02370
CAR16	Slangenbaai	N 12°08.359′	W 068°59.840′	10	52	0.02624
CAR17	Habitat Curaçao	N 12°11.877′	W 069°04.751′	10	68	0.02484
CAR18	Daaibooi	N 12°12.710′	W 069°05.087′	9	47	0.02486
CAR19	PortoMari	N 12°13.103′	W 069°05.153′	8	44	0.02466
CAR20	Marie Pompoen	N 12°05.459′	W 68°54.338′	2	14	0.02074
CAR21	W Piscadera Bay	N 12°07.464′	W 68°58.288′	5	33	0.02400
CAR22	Playa Largu	N 12°14.118′	W 69°05.907′	10	58	0.02808
CAR23	Boka Pos Spano	N 12°16.700′	W 69°08.591′	14	52	0.02204
CAR24	Diver’s Leap	N 12°04.439′	W 68°52.714′	2	22	0.03259
CAR25	Playa Forti	N 12°21.976′	W 69°09.220′	6	45	0.03032
CAR26	Grote Knip	N 12°21.087′	W 69°09.095′	18	63	0.02453
CAR27	Tugboat	N 12°04.152′	W 68°51.708′	8	36	0.02127
CAR28	Slangenbaai	N 12°03.797′	W 68°51.190′	9	56	0.02782
CAR29	Fuikbaai	N 12°02.855′	W 68°49.838′	9	47	0.02564
CAO17	Cas Abou	N 12°13.435′	W 69°05.304′	3	23	0.02272
