# Supplementary material for: Extreme mitochondrial variation in the Atlantic gall crab Opecarcinus hypostegus (Decapoda: Cryptochiridae) reveals adaptive genetic divergence over Agaricia coral hosts
Source: Sci Rep. 2017 Jan 12;7:39461. doi: 10.1038/srep39461 (PMC5228066; doi:10.1038/srep39461)
Supplement: Supplementary Fig. S1 [file srep39461-s1.pdf]

Supplementary information belonging to manuscript

Extreme mitochondrial variation in the Atlantic gall crab *Opecarcinus hypostegus* (Decapoda: Cryptochiridae) reveals adaptive genetic divergence over *Agaricia* coral hosts

Kaj M. van Tienderen[1], Sancia E.T. van der Meij[1,2,3*]

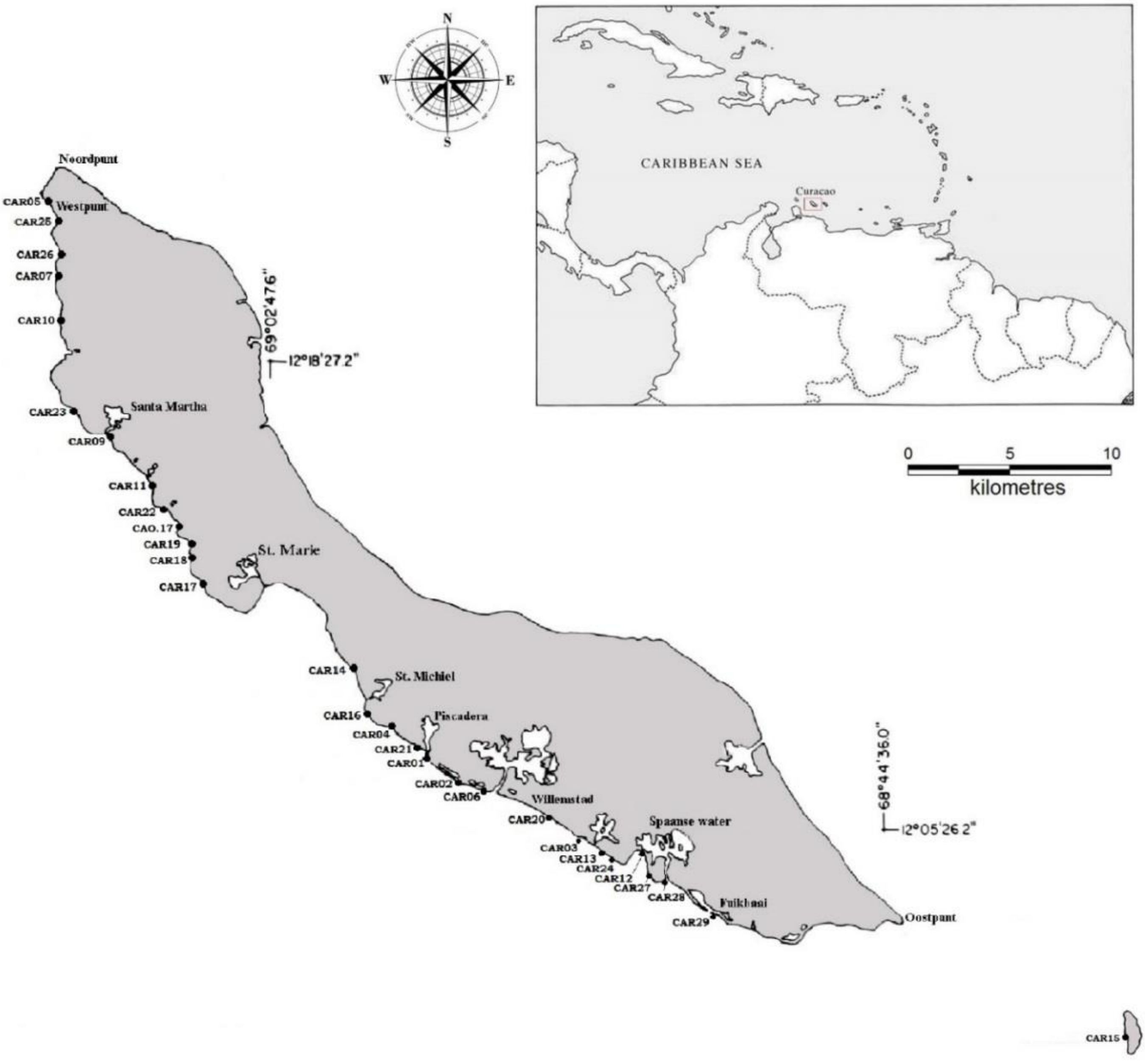

Fig. S1. Sampling localities along the leeward coast off Curaçao, adapted from Van Tienderen & Van der Meij (2016: Fig. 2). Detailed locality data is provided in Table 1.

Reference

van Tienderen, K.M. & van der Meij, S.E.T. Occurrence patterns of coral-dwelling gall crabs (Cryptochiridae) over depth intervals in the Caribbean. *PeerJ* **4**, e1794 (2016).

Captions separate Excel files

Table S1. Collection data and GenBank accession numbers for the material used in this study.

Table S2. Genetic Distance (Pairwise Φst) between localities.

Table S3. Geographical distance (km) between localities.
